# Developing Combined HIV Vaccine Strategies for a Functional Cure

**DOI:** 10.3390/vaccines1040481

**Published:** 2013-10-28

**Authors:** Alessandra Noto, Lydie Trautmann

**Affiliations:** Vaccine and Gene Therapy Institute of Florida, Port Saint Lucie, Florida, FL 34987, USA

**Keywords:** HIV, therapeutic vaccine, CD8^+^ T cells, reactivation of viral reservoirs, combination therapy, functional cure

## Abstract

Increasing numbers of HIV-infected individuals have access to potent antiretroviral drugs that control viral replication and decrease the risk of transmission. However, there is no cure for HIV and new strategies have to be developed to reach an eradication of the virus or a natural control of viral replication in the absence of drugs (functional cure). Therapeutic vaccines against HIV have been evaluated in many trials over the last 20 years and important knowledge has been gained from these trials. However, the major obstacle to HIV eradication is the persistence of latent proviral reservoirs. Different molecules are currently tested in ART-treated subjects to reactivate these latent reservoirs. Such anti-latency agents should be combined with a vaccination regimen in order to control or eradicate reactivated latently-infected cells. New *in vitro* assays should also be developed to assess the success of tested therapeutic vaccines by measuring the immune-mediated killing of replication-competent HIV reservoir cells. This review provides an overview of the current strategies to combine HIV vaccines with anti-latency agents that could act as adjuvant on the vaccine-induced immune response as well as new tools to assess the efficacy of these approaches.

## 1. Introduction

Despite the undeniable success of antiretroviral therapy (ART) in limiting HIV replication, it has become increasingly evident that ART is not a long-term solution for HIV-infected individuals. Besides the deleterious side effects, ART does not eradicate HIV and does not optimally reconstitute the immune system [1,2]. Novel immunotherapeutic strategies that would induce the immune-mediated control of HIV replication in the absence of ART (also called “functional cure”) are needed. However, tested vaccination strategies have so far shown limited success (reviewed in [3]). Immune mechanisms of HIV control are still unknown and need to be elucidated in order to help develop these therapeutic interventions. In individuals who naturally maintain undetectable viral load without ART, called elite controllers (ECs), the control of HIV replication in the absence of treatment has been primarily attributed to an effective adaptive immune response [4]. However, in most HIV-infected subjects, HIV-mediated immune damages (mainly CD4^+^ T cell depletion and chronic inflammation) lead to a dysfunctional immune response that is not restored by ART [1,5]. Moreover, viral control is only achieved by ART and treatment interruption results in a rapid return of viremia. 

The major obstacle to HIV eradication is the persistence of latent proviral reservoirs that are not targeted by antiretroviral regimens [6,7,8,9,10]. Eradication strategy aims at the induction of viral replication in latently-infected cells and at the elimination of these reactivated cells by either direct cytolytic targeting or by immunotherapeutic intervention [11]. HIV-specific CD8^+^ cytotoxic T cells (CTLs) are important for the control of HIV replication in non-treated individuals but in subjects under ART their number is too low to kill the latent reservoirs. Recently, Dr. Siliciano’s group showed that after *in vitro* expansion of HIV-specific CD8^+^ T cells from ART-treated subjects, these cells were able to eliminate HIV-infected CD4^+^ T cells [12]. This seminal study provided the rationale for new therapeutic strategies that combine agents that reactivate latently-infected CD4^+^ T cells with immune interventions that increase the numbers and function of HIV-specific CD8^+^ CTLs to clear HIV reservoirs in individuals on ART. However peptide-stimulated HIV-1-specific CD8^+^ T cells were used in this study, which might not reflect *in vivo* approaches. In addition, in this study only one histone deacetylase inhibitor (HDACi), Saha, was used. This molecule was also used in a clinical trial and was able to increase the levels of HIV-DNA in ART-treated donors [13]. Other more potent HDACis may induce stronger cytotoxic effects during HIV-1 reactivation, and cause selective cell death in CD4^+^ T cells in which successful viral reactivation occurs. Different approaches are currently being tested to reactivate latently-infected cells and restore immune functions. These molecules include HDAC inhibitors, mediators of T cell homeostasis or antibodies to block negative regulators; they all focus on reactivating latently-infected CD4^+^ T cells to render them susceptible to immune-mediated killing while also potentiating HIV-specific CTLs to kill reactivated CD4^+^ T cells. This review will describe the current knowledge and advances using these therapeutic strategies.

## 2. CD8 T Cell-Based Vaccine Strategies for a Functional Cure

Functional cure from HIV infection was achieved for the first time by Timothy Brown, the Berlin patient, who was given hematopoietic stem cell transplant (HSC) from CCR5 delta 32 donor (mutation of the gene required for HIV entry) [14]. Brown remains HIV free without ART after 6 years. Recently, two subjects from Boston, USA, with Hodgkin’s lymphoma and treated with ART were given a CCR5^+/+^ haemopoietic stem-cell transplant [15]. The two subjects had undetectable HIV-DNA years after transplantation. These findings suggest that ablative conditioning, immunosuppressive treatment, and post-transplant graft-versus host might be the reasons of the functional cure, more than the CCR5 deletion.

The components of an efficient immune response able to control HIV replication after treatment interruption are still to be identified. In ECs, the natural control of HIV replication has been mainly attributed to strong T cell responses directed to dominant epitopes restricted by HLA types associated with viral control [4,16,17]. However, these responses cannot be elicited by a vaccine regimen in subjects that do not carry these HLAs. Recent studies suggested that initiating ART in early HIV infection could lead to control of HIV replication control in 5% to 15% of individuals after analytical treatment interruption (ATI) in individuals missing the known genetic characteristics of ECs [18,19,20,21,22,23,24,25,26,27]. In the VISCONTI cohort, 14 subjects achieved long-term post-treatment control of HIV replication after ART cessation [18,21]. Very early treatment (30 h after birth) was given to the Mississippi baby that was the first case of functional cure of an infant [19]. What cured the baby is still not fully understood but one reason might be prevention of the formation of latent reservoirs by very early treatment. These rare cases initiated ART during the early/acute phase of infection and interrupted ART after some years of ART, suggesting that early treatment initiation in acute HIV infection would lead to specific immune functions that are able to control HIV replication after ART cessation. Early and prolonged ART has been recently shown to be associated with an HIV-specific CD8^+^ T cell cytokine profile comparable to that of long-term non-progressors [28,29]. However, the mechanisms of viral control in this small number of subjects have not been elucidated yet. Furthermore, the magnitude of HIV-specific CD8^+^ T cells is low in these very early treated donors, which may limit studies. Moreover, no evidence of HIV-specific CD8^+^ T cells-mediated control of viral control after ART cessation was demonstrated in the VISCONTI cohort [21]. Other studies are currently testing whether the very early treatment in acute infection could lead to control of viral rebound after ATI [30,31]. The analysis of the characteristics of the immune response directed against HIV before and after ART interruption in these rare cases will provide clues that will guide the development of new vaccine interventions. 

Several studies suggest the need for efficient HIV-specific CD8^+^ T cells to control viral replication after treatment cessation. HIV-specific CD8^+^ T cells are a promising tool to eliminate reactivated, latently-infected CD4^+^ T cells and cure HIV infection as they are already important for the control of HIV-1 replication in non-treated individuals [16,32]. However, upon initiation of ART, their frequency declines rapidly and few memory cells are maintained. Previous efforts aimed at augmenting HIV-specific CD8^+^ T cell responses with structured treatment interruptions or vaccine regimens in ART-treated donors and in non-human primates have not been successful to control viral replication after ART cessation. Recently, Hansen *et al.* described a new RhesusCMV viral vector that induced the viral control of half of the infected macaques after challenge [33]. The wide breadth and non-conventional CD8^+^ T cell responses induced by the vaccine could contribute to the dramatic control of SIV replication in the vaccinated animals [33]. These results suggest that different and more efficient CD8^+^ T cell responses targeting HIV epitopes could control HIV replication. 

HIV-specific CD8^+^ T cells play a crucial role in mediating antiviral immunity by killing the productively infected CD4^+^ T cells. The critical role of CD8^+^ T cells in controlling viral replication has been demonstrated in acute infection in the SIV model, where CD8^+^ T cells depletion leads to a sharp increase in viremia [34]. Several other observations suggest that HIV-specific CD8^+^ T cells are important for the control of HIV-1 replication, including the generation and maintenance of viral escape mutations in CTL epitopes or the superior control of viral replication by certain HIV-specific clonotypes restricted by HLA-B57 and B27 [16]. Yang *et al.* demonstrated a significant association between CD8^+^ T cell viral inhibition activity *in vitro* and the rate of CD4^+^ T cell loss in early HIV infection and CD4^+^ T cell decline in chronically infected individuals [35]. In a macaque model for HLA-B27 mediated viral control, the control of viral replication was associated with high frequencies of SIV-specific CD8^+^ T cell responses directed against three epitopes [36]. These data highlight the important role of eliciting efficient CD8^+^ T cells for the control of viral replication [23]. Previous reports have shown that *in vivo* induction of high-avidity, high-frequency CD8^+^ T cell responses were associated with antiviral protective immunity [36,37,38]. The selection and maintenance of high affinity clonotypes early in acute infection could play an important role in the efficient killing of infected CD4^+^ T cells after treatment interruption [16,39,40]. However, clonal depletion has been observed during primary HIV infection [41]. We have previously shown a marked degree of clonotypic turnover within HIV-specific CD8^+^ T cell populations as a consequence of antigen decay after the initiation of ART with particular clonotypes selected for their higher functional sensitivity [42]. Therefore, maturation of the TCR repertoire towards these high affinity clonotypes could play an important role in viral control after ATI. However, previous efforts aimed at augmenting CD8^+^ T cell responses in ART-treated donors with structured treatment interruption or using vaccine regimens have not been as successful as hoped to control viral replication after ART cessation [24,43,44,45,46]. Novel CD8 T cell-based vaccine strategies are therefore needed to increase the number and function of HIV-specific CD8^+^ T cells that could control viral rebound after ART cessation, with the ultimate goal of achieving spontaneous control of viral replication without treatment [47]. 

## 3. “Shock and Kill” Strategy

Several molecules currently tested in clinical trials to reactivate HIV from latently-infected CD4^+^ T cells have demonstrated promising *in vitro* and *ex vivo* activities in reactivating latent HIV reservoir (shock) [48,49,50,51]. These molecules include HDAC inhibitors, mediators of T cell homeostasis or antibodies to block negative regulators. These molecules could also differentially influence the selection, expansion, persistence and function of HIV-specific CD8^+^ T cell responses stimulated contemporaneously by a vaccine strategy (kill).

### 3.1. HDAC Inhibitors

Post-translational modification including phosphorylation, acetylation, methylation and ubiquitination are thought to contribute to transcriptional regulation by inducing an “open” (transcriptionally permissive) *vs*. “closed” (transcriptionally repressive) state of chromatin. During HIV infection, many transcription factors bind to the LTR and induce silencing of the HIV promoter by recruiting histone deacetylases (HDACs). C-promoter binding factor-1 (CBF-1) and the NF-kappaB homodimer p50/p50 are two of the transcription factors that have been shown to bind the LTR enhancer sequence thus promoting transcriptional silencing during the establishment of HIV-1 latency [52,53]. Thus, one of the promising candidate families of molecules tested in clinical trials to reactivate latent HIV reservoirs are histone deacetylase inhibitors (HDACis). Among the HDACis, valproic acid (VPA) and suberoylanilide hydroxamic acid (SAHA) have been tested in HIV infected subjects under ART. However in the first clinical trial, VPA failed to reactivate the virus from latently infected cells and thus to decrease the reservoir. Most promising results in a recent clinical trial showed that SAHA was able to reactivate viral reservoirs in subjects on ART [13]. There is limited knowledge of the chromatin remodeling and non-epigenetic effects of HDACis on the differentiation of CD8^+^ T cells in humans. Studies in mice have shown that CD8^+^ T cells activated without CD4^+^ help, fail to develop functional, protective memory and remain hypo-acetylated. Treatment with an HDACis increased histone acetylation in unaided CD8^+^ T cells and restored their ability to differentiate into functional memory cells capable of immediate cytokine production and providing protective immunity [54]. Besides being able to reactivate viral production in latently-infected CD4^+^ T cells and increase HIV antigen presentation by MHC I, HDACis could also change the fate of HIV-specific CD8^+^ T cells induced by a vaccine.

### 3.2. Targeting Negative Regulators

The combination of systems biology, phenotypic and functional profiles suggests that PD-1 is a good target for therapeutic interventions aimed at restoring CD8^+^ T cell function in HIV infection. Different groups have already shown that a PD-1 blockade in various diseases such as HIV, hepatitis B, and hepatitis C is able to restore T cell proliferation, cytokine production and thus effector function [55,56,57,58,59,60,61]. While it has been suggested that blocking PD-L1 has a better capacity to restore T cell function than targeting PD-1 itself [55,62], in both cases blocking this interaction resulted in increased HIV-specific CD8^+^ T cell proliferation [63]. Recent *in vivo* studies have been conducted in the rhesus macaque SIV infection model [58,64]. Velu *et al.* showed that PD-1 blockade resulted in increased frequencies of SIV specific CD8^+^ T cells, increased cytotoxic function and decreased viral load [58]. Many groups have also shown that the exhaustion of HIV specific CD8^+^ T cells from chronic HIV infected subjects is associated with the co-expression of different negative regulators, such as PD-1, CD160 and 2B4. The most exhausted cells simultaneously express multiple negative regulatory receptors on their cell surface and their expression positively correlates with viral load and decreased cytokine production [65,66]. Blocking the interaction of CD160 and HVEM was able to enhance and rescue HIV-specific CD8^+^ T cell proliferation and cytokine production. All together, these findings are in agreement with previous studies that provide a strong rationale for initiating human clinical trials targeting PD-1 with blocking antibodies in HIV-infected subjects. Blocking PD-1 or multiple negative regulators might not only increase effector functions of exhausted HIV-specific CD8^+^ T cells but could also reactivate the viral reservoir from latently infected CD4^+^ T cells [67]. Thus, combination therapy that combines vaccination under ART with blocking antibodies for PD-1 signaling might prove to be more potent in increasing CD8^+^ T cell killing of latently infected cells and to preventing reactivated virus from re-infecting new CD4^+^ T cells. 

### 3.3. Gamma Chain Cytokines

Gamma chain cytokines (IL-2, IL-15, IL-7 and IL-21) that converge on the STAT5A/B signaling pathway have been considered as candidates to regulate reservoir reactivation. These cytokines can also be used as modulators of a vaccine immune therapy [68]. During HIV infection, production of some of these cytokines, such as IL-2 and IL-15, is downregulated, while IL-7 levels are increased as a consequence of lymphopenia. Extensive phase I and II studies were done in the late 1990s with IL-2 as a candidate cytokine for treatment of subjects with HIV infection. These studies demonstrated that this cytokine increases the frequency of naïve and central memory (T_CM_) CD4^+^ T cells, as well as CD8^+^ cytotoxic functions [69,70,71]. However, phase III clinical trials demonstrated that IL-2 increased CD25 and FOXOp3 expression (and thus regulatory T cells), which was associated with increased risk of opportunistic diseases [71,72,73]. Similar to IL-2, IL-15 signals through the IL-2RB (CD122) and γ-chain (CD132) receptors and plays an essential role in T cell survival [74]. Although both IL-2 and IL-15 induce identical signal-transduction pathways and proliferative responses in T cells and NK cells, IL-2 favors maintenance of peripheral regulatory T cells and participates in activation-induced cell death while IL-15 preferentially stimulates expansion of CD8^+^ T cells, NK, and NKT cells [75]. Stimulation of cells from HIV-infected subjects with IL-15, enhances the frequency of effector memory CD8^+^ T cell, promotes their survival and cytotoxic functions [76,77,78,79,80]. Stimulation with IL-15 might also relieve HIV-specific CD8^+^ T cells from their functional and phenotypic block in a transitional memory (T_TM_) phenotype (CCR7^−^, CD27^+^, CD45RA^−^) and induce their differentiation in functional effector antigen-specific CD8^+^ T cells [67]. Preclinical studies have shown that IL-15 enhances CTL responses in murine tumor models and the administration of IL-15 in combination with anti-PD-L1and anti-CTLA-4 antibodies showed greater cytolytic and effector functions on the CD8^+^ T cells of metastatic tumor-bearing animals [81,82,83,84,85]. Studies in non-human primates showed that daily administration of IL-15 was not only safe but increased the frequency of effector memory (T_EM_) CD8^+^ T cells of 100-fold [86,87]. However another study showed that administration of IL-15 in chronically SIV infected macaques, in combination with ART, resulted in a delay in viral suppression and failed in the reconstitution of CD4^+^ T cell numbers after ART interruptions [88]. 

IL-7 regulates T-cell maturation and supports peripheral T cell homeostasis. Preclinical studies in SIV-infected macaques showed that IL-7 injection was not toxic and was not increasing viremia [89,90]. Phase I/II trials in HIV infected subjects with persistent lymphopenia have demonstrated that the administration of IL-7 was able to restore circulating CD4 T-cell counts as well as the frequency of CD8^+^ T cells, mainly those with a central memory (T_CM_) phenotype [91] without any effect on the number of regulatory T cells. IL-21 is another γ-chain cytokine that induces a potent cytotoxic activity of NK and CD8^+^ T cells from HIV infected subjects and promotes antiviral activity in human CD8^+^ T cells [92]. HIV-specific IL-21^+^ CD4^+^ T cell responses contribute to durable viral control through the modulation of HIV-specific CD8^+^ T cell function [93]. The benefic role of this cytokine has been confirmed in a pilot study where IL-21 was injected in SIV infected rhesus macaques in late-stage disease [94]. Administration of IL-21 resulted in increased cytotoxic effector molecules in CD8^+^ T cells and NK cells and as well as enhanced B cell differentiation.

Eradication strategies should rely not only in boosting CTL activity, but also at enhancing HIV reactivation from latently infected CD4^+^ T cells. Few studies have shown that among the γ-chain cytokines, IL-7 is a potent and proviral strain-specific inducer of latent HIV-1 cellular reservoirs [50,95]. Vandergeten *et al.* showed that IL-7 increases reactivation in productively infected cells but has no effect in latently infected CD4^+^ T cells whereas IL-15 seems to be a more potent inducer of viral production from latently infected CD4^+^ T cells than IL-7 [96,97]. Administration of IL-15 during the contraction phase (7–14 days) and not during the expansion phase could have a better effect in the enhancement of HIV-specific CD8^+^ T cells response after vaccination. Further studies should be done to determine the timing of cytokine delivery in combination with a vaccine regimen. All together these findings provide a good rationale for complementing vaccine therapy with γ-chain cytokines in eradication strategies aimed at purging latent HIV from CD4^+^ T cells and at boosting HIV-specific CD8^+^ T cell responses.

## 4. New Assays to Monitor an Effective Combined Therapy for a Functional Cure

Few parameters including frequency, phenotype, and cytokine production are commonly used to assess T cell responses [98]. The frequency and phenotype of T cells are limited in evaluating their functions. T cell poly-functionality has been suggested to correlate with HIV disease control, however it is still unclear if this feature is sufficient to provide T cell-mediated immune control [99,100]. Therefore, new functional assays have to be developed to measure T cell functions focusing on select immune parameters known to impact the control of HIV replication. The cytolytic function of CD8^+^ T cells is critical for eradicating HIV-infected CD4^+^ T cells and control viral replication after ART interruption. New *in vitro* methods to evaluate the viral replication inhibition by CD8^+^ T cells have been recently developed [12,101,102] and can be used to recapitulate the *in vivo* cytotoxic activity of CD8^+^ T cells after ATI. A study by Yang *et al.* demonstrated a significant association between CD8^+^ T cell antiviral activity *in vitro* and the rate of loss in early HIV-1 infection and CD4^+^ T cell decline in chronically infected individuals using an *in vitro* viral inhibition assay [35]. We developed a new assay to quantify the intrinsic killing capacity of HIV-specific CD8^+^ T cells measured in lytic units [103]. Using this assay, we have observed that HIV-specific CD8^+^ T cells in primary infection exhibited a significantly higher cytotoxic capacity than HIV-specific CD8^+^ T cells in chronic infection [104]. These new functional cell-based assays can help determining if an enhanced cytotoxic activity of CD8^+^ T cells is associated with viral control after ATI. Furthermore, new assays that can recapitulate the killing of primary latently-infected CD4^+^ T cells by HIV-specific CTLs from ART treated donors would be an important platform to test the immune intervention strategies of reservoir eradication as it would provide the direct readout of elimination of reactivated latently-infected cells. 

## 5. Conclusions

New CD8^+^ T cell-based vaccination strategies under ART should be evaluated in combination with reactivating agents to achieve control of viral replication upon ART interruption. Immunotherapeutic interventions not only need to increase the number of HIV-specific CD8^+^ T cells *in vivo* but also induce efficient cytotoxic CD8^+^ T cells to kill reactivated latently-infected CD4 T cells. However, the mechanisms that underlie such successful immunotherapies remain unknown. These immune interventions have to be combined with contemporaneous strategies aimed at reactivation of the latent HIV reservoir. Combinations could also include new strategies such as vaccination with a biologically active Tat protein that protected non-human primates against an R5-SHIV challenge by neutralizing antibodies [105]. These combinations have to be judiciously selected to potentiate the vaccine-induced response. The effect of reactivating agents on the immune response induced by therapeutic interventions has to be also evaluated. The accurate assessment of new therapeutic vaccine regimen would accelerate the development of successful therapies for a functional cure.
